# Everybody's Page

**Published:** 1890-04-19

**Authors:** 


### Everybody's Page.

Many of the poor Japanese who earn a living by running the "Jinrikishas" or man-carriages fall victims to heart disease.

Those who like it, will find chocolate in the solid form very sustaining. On long walks, out hunting, or even climbing, a good-sized round or oblong piece of chocolate will go a long way towards refreshing tired nature and stopping a premature longing for the sound of the dinner-bell.

There is said to be a plant in Arabia, with flowers of bright yellow and with seeds which are like black beans, and these dried and powdered, and taken in small doses, cause a person to dance about and behave like a lunatic, till he becomes exhausted and falls asleep. When he awakes, he has not the smallest remembrance of his ridiculous behaviour. The plant is called a "laughing-plant," but where the laugh comes in it is hard to say.

Chinese doctors are very particular about the distinction being very strictly kept up between physicians and surgeons, and would not trespass on one another's ground for the world, but this delicacy of feeling has rather a disastrous effect on the patients' pockets sometimes. A Chinese gentleman was struck by an arrow, which remained fast in his body. A surgeon was sent for, and modestly requesting his fee should be paid in advance, he broke off the protruding bit of the arrow, leaving the point imbedded in the wretched man's body. He refused to extract it, because, said he, medical etiquette forbids it; the case is clearly one for a physician, since the arrow is *inside* the body!

The agriculturist, if he believes in the principle that "every little helps," has an ally in the glow-worm, for as a grub it feeds entirely upon snails, and every living glow-worm has been said to represent three dead snails.

Mr. Bent in the February *Fortnightly*, described the method of curing fever in Nicæa, the city of the creed; it boasts simplicity, and is, we should say, unique. A shirt of the fevered man must be carried to the priest, and a bottle of water must be blessed in a dervish's bowl. The sick man puts on the shirt and drinks of the water, then if he can walk, he goes and lies down before the Dervish, who steps upon him. If he dies, fate, stern fate (not the Dervish's weight) has caused his death, and if he recovers, then religion has come to his rescue. Little children and babies are treated in precisely the same manner.

Quack doctors will have to mind their manners in Oregon, U.S.A., or else find a fresh field to practise in, for the Legislature is making a law to regulate the practice of medicine, which contains a clause against "unprofessional conduct." Amongst the actions so defined in the Bill it will be illegal to take a fee on the assurance that an incurable disease can be permanently cured, or to disclose a professional secret, or to advertise medical business in which improbable or untruthful statements are made. Habitual intemperance will also be a breach of the law. These stringent measures will tax the ingenuity of the gentlemen who gain a living by advertising and selling wondrous medicines capable of curing any disease under the sun.

